# Effect of Hemp Extraction Procedures on Cannabinoid and Terpenoid Composition

**DOI:** 10.3390/plants13162222

**Published:** 2024-08-10

**Authors:** Francisco T. Chacon, Wesley M. Raup-Konsavage, Kent E. Vrana, Joshua J. Kellogg

**Affiliations:** 1 Intercollege Graduate Degree Program in Plant Biology, Pennsylvania State University, University Park, State College, PA 16802, USA; ftc5040@psu.edu; 2 Department of Pharmacology, Penn State College of Medicine, Hershey, PA 17033, USA; wkonsavage@pennstatehealth.psu.edu (W.M.R.-K.); kev10@psu.edu (K.E.V.); 3 Department of Veterinary and Biomedical Sciences, Pennsylvania State University, University Park, State College, PA 16802, USA

**Keywords:** extraction, cannabinoids, cannabis, terpenes, supercritical, hydrodistillation, ethanol extraction

## Abstract

A variety of techniques have been developed to extract hemp phytochemicals for research and consumption. Some of the most common processes in the industry include supercritical CO_2_ extraction, hydrodistillation, and solvent-based (ethanol) extractions. Each of these processes has the potential to differentially extract various phytochemicals, which would impact their efficacy, tolerability, and safety. However, despite these differences, there has been no direct comparison of the methods and the resulting phytochemical composition. This work aimed to compare cannabinoid and terpene profiles using the three primary commercial procedures, using hemp inflorescence from a CBD/CBG dominant *Cannabis sativa* L. cultivar. Extracts were then evaluated for their terpene and cannabinoid content using GC-MS and LC-MS/MS, respectively. Hydrodistilled extracts contained the most variety and abundance of terpenes with β-caryophyllene to be the most concentrated terpene (25–42 mg/g). Supercritical CO_2_ extracts displayed a minimal variety of terpenes, but the most variety and abundance of cannabinoids with CBD ranging from 12.8–20.6 mg/g. Ethanol extracts contained the most acidic cannabinoids with 3.2–4.1 mg/g of CBDA along with minor terpene levels. The resulting extracts demonstrated substantially different chemical profiles and highlight how the process used to extract hemp can play a large role in product composition and potential biological effects.

## 1. Introduction

Cannabinoids and terpenoids are widely recognized as the primary therapeutic agents of hemp (*Cannabis sativa* L.) inflorescence and extracts [1,2]. These phytochemical families can be found in copious amounts in the trichrome structures of female hemp inflorescence, and in smaller quantities within the leaves [3]. Cannabinoids are a group of compounds that contain a C32 terpenophenolic backbone, derived from the condensation of hexanoyl-CoA and three malonyl-CoA molecules and undergoing multiple cyclization and aromatization to produce myriad structures (>100 cannabinoids are currently known) [4,5,6,7,8,9,10]. Similarly, cannabis contains a large diversity of terpenoid structures, due to the wide biosynthetic capacity of the plant; there are >30 cannabis terpene synthases (CsTPS) genes identified (yet only 9 are fully characterized) [11,12,13]. Originating from multiple condensation reactions of the 5-carbon precursor isopentenyl diphosphate, terpenoids are classified according to the number of isoprene units (e.g., monoterpenes (two isoprene units), sesquiterpenes (three isoprene units), and diterpenes (four isoprene units) [1,11,14,15,16,17,18,19].

Hemp products, consisting of various cannabinoids and terpenoid profiles, have grown exponentially as the plant has been more widely accepted and legalized and the benefits have become more apparent [20,21]. A commercial hemp product’s unique biological activity or flavor is determined by its levels of cannabinoids/terpenoids, which in turn is influenced by a number of variables, including strain differences, growing conditions, post-harvest practices, or the extraction processes used [22,23,24,25,26,27]. However, the selection of extraction process can ultimately lead to more diversity and abundance of specific chemical constituents based on compound properties, providing industries and researchers a direct approach to harnessing the biological effects of cannabis compounds.

There are a number of extraction techniques that have been developed for the extraction of phytochemicals from hemp inflorescence; the most prominent in the industry include supercritical fluid CO_2_ extraction (SFE), solvent-based extractions, hydrodistillation, ultrasonication-assisted, and microwave-assisted extraction [28,29,30,31,32]. Supercritical fluid extraction is among the most popular extraction techniques due to its high specificity, lack of organic solvents, yield capacity, and extraction time [32,33]. SFE technique often utilizes CO_2_ in a supercritical fluid state by creating high pressure and high temperature conditions that permits the separation of non-polar compounds [34]. Organic solvent extraction methods (including ethanol, butanol, and isopropyl extractions) vary as the phytochemicals they extract depend on the solvent polarity and hydrogen bonding characteristics [35]. Solvent extractions are a primary method of producing botanical supplements and have proven to be a cost-effective approach for the extraction of cannabinoids; however, they may result in a fuller-spectrum extract, an extraction of all chemical constituents within the plant matrix depending on temperature, lack of winterization, or decarboxylation [31,36]. Hydrodistillation is a third technique that allows for the collection of aromatic compounds from plant material, often used when producing “essential oils” [37]. This utilizes a distillation apparatus that separates the essential oil from condensed vapor obtained from a boiling mixture of water and plant material. Other extraction approaches have been investigated, such as eutectic solvents, pulsed electric fields, and hydrodynamic extractions [38,39,40].

Given the popularity of SFE, hydrodistillation, and solvent-based extractions in the cannabis industry; there have been several studies that have examined the production of cannabinoids and terpenoids when employing these extraction techniques. Including the investigation by Mazzara et al., 2022 analyzed terpene, polyphenol, and cannabinoid of hydrodistilled extracts from nine commercial varieties of *Cannabis sativa* L. [41]. Providing evidence of how any hemp cultivar when subjected to hydrodistillation will result in copious amounts of terpenes as much as 30.2% of total concentration. Likewise, in 2021 Bowen et al., reported the impact of solvent-based extraction (ethanol or isopropyl alcohol) and super critical CO_2_ on the chemical profile of flower from single cannabis cultivar [36]. Interestingly, more neutral cannabinoids were detected in solvent-based extracts and more acidic cannabinoids in CO_2_ extracts, an outcome believed to be influenced by the timing of decarboxylation.

However, thus far no study has directly compared the terpene and cannabinoid content of the three dominant extraction techniques from the same botanical material. The present investigation compared three hemp extraction techniques frequently seen in commercial applications: aqueous ethanol maceration, hydrodistillation, and supercritical fluid extraction. One hemp cultivar, grown in three different geographical locations, was harvested and processed identically with the resulting extracts analyzed for their targeted chemical profiles and quantification of known bioactive compounds. This study provides direct evidence that the extraction method yields distinct phytochemical profiles and could be employed to obtain more selective cannabinoid or terpene chemical profiles. 

## 2. Results

### 2.1. Quantification of Terpenes, Cannabinoids, and Extraction Yield

**Figure 1:**
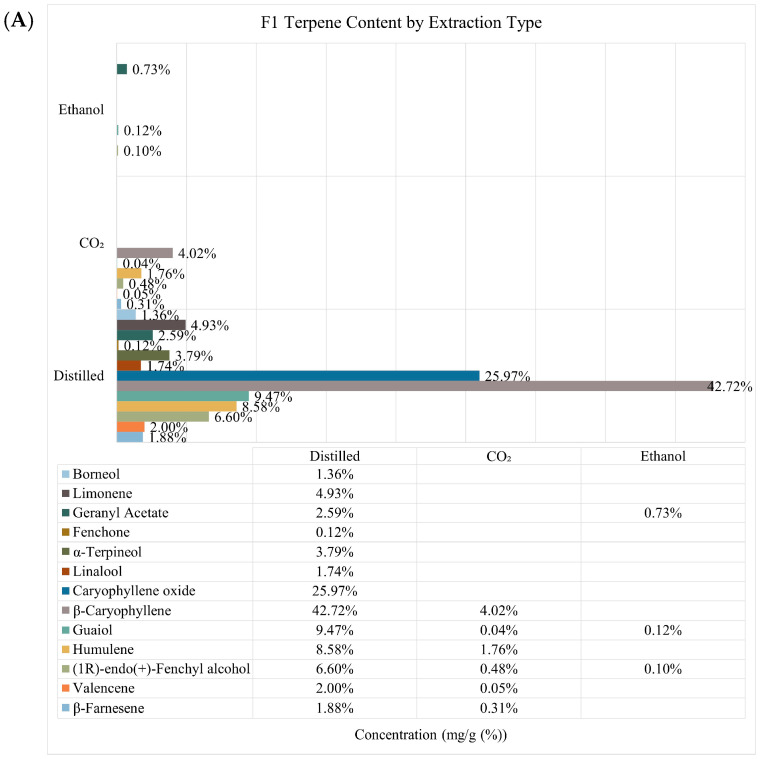

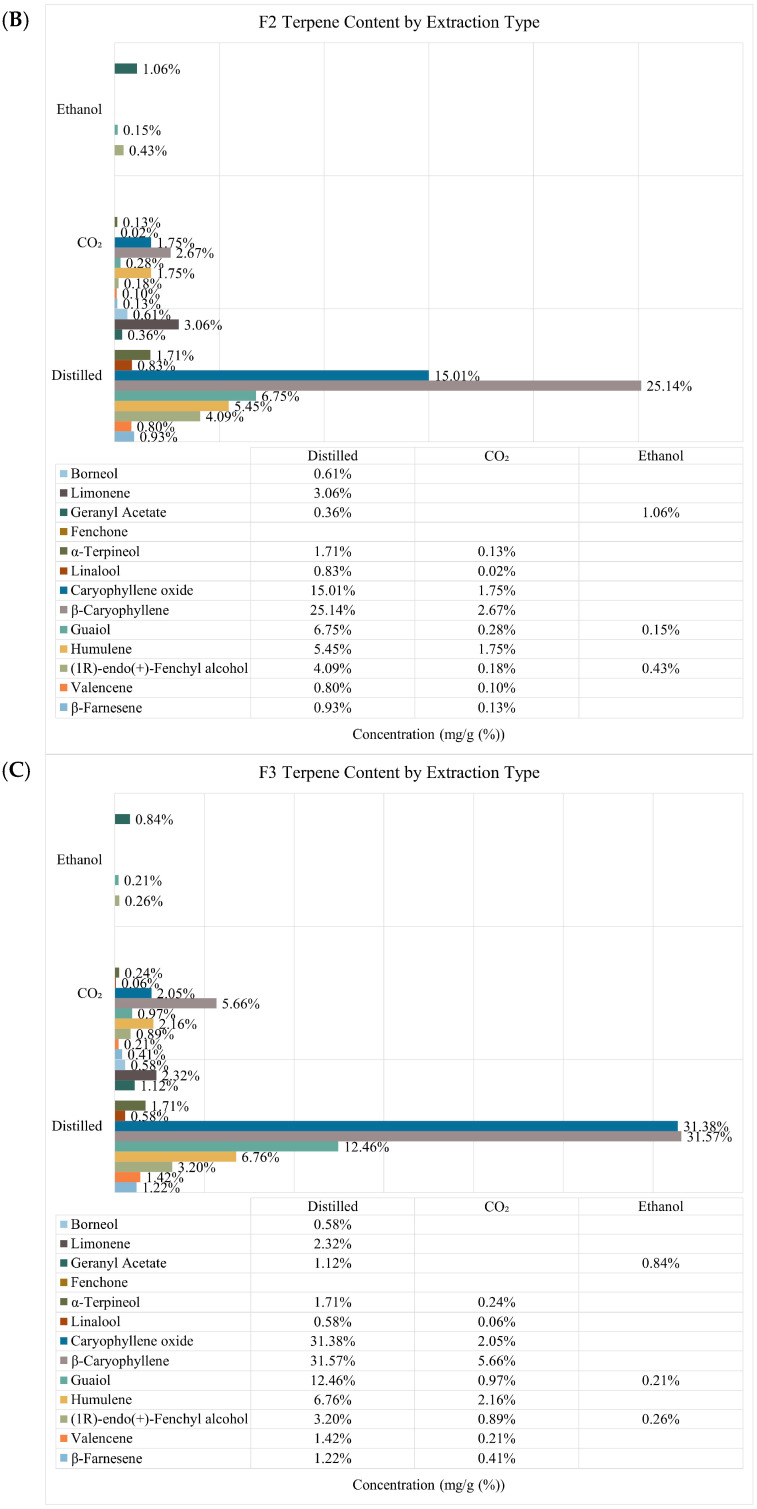
Relative composition of identified terpene concentration from each extraction method. Data labels indicate absolute quantitation (mg/g (%)) of each compound in the corresponding extract. (**A**) F1 samples, (**B**) F2 samples, and (**C**) F3 samples.

**Figure 2:**
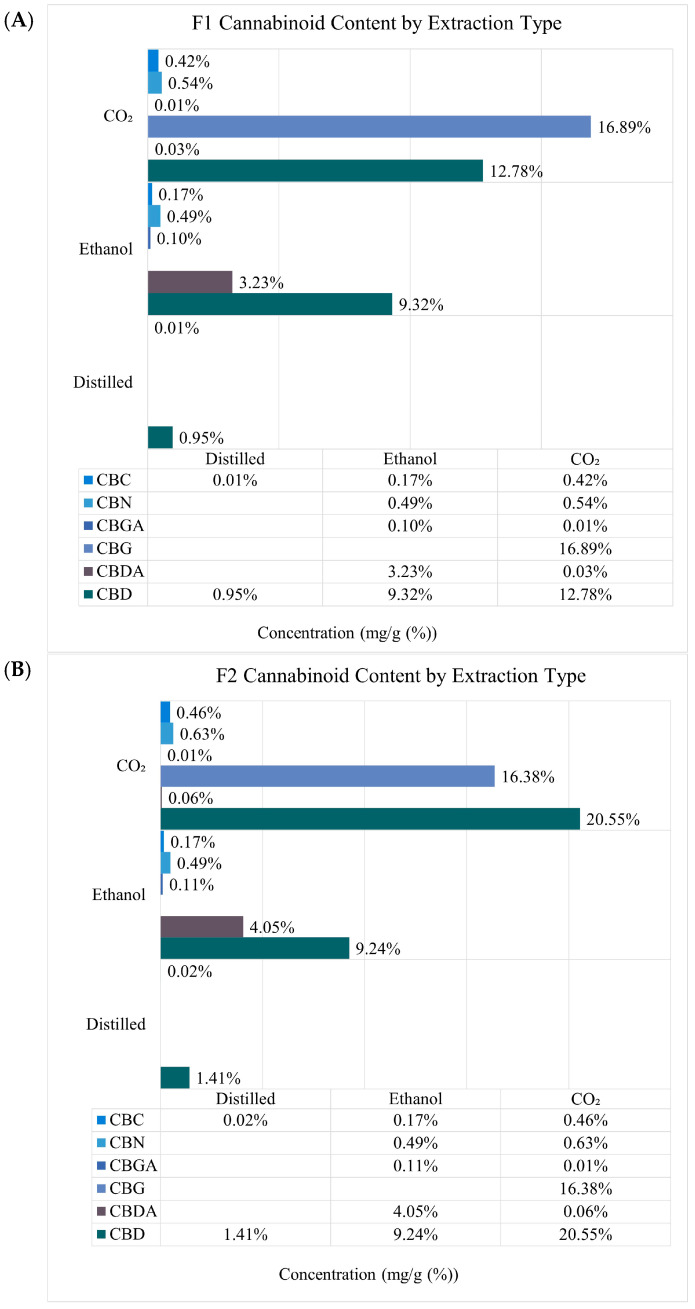

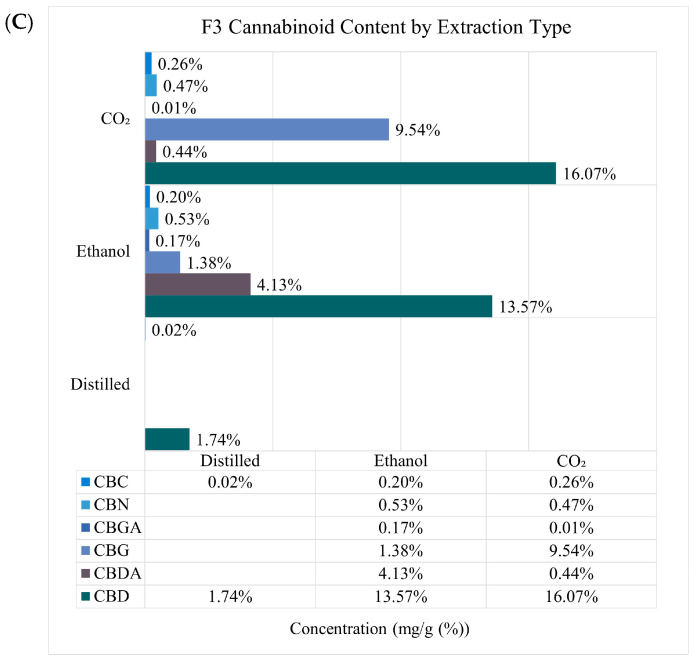
Relative composition of identified cannabinoid concentration from each extraction method. Data labels indicate absolute quantitation (mg/g (%)) of each compound in the corresponding extract. (**A**) F1 samples, (**B**) F2 samples, and (**C**) F3 samples.

Terpene concentration varied widely depending upon the hemp sample and extraction type. Hydrodistilled samples, displayed the most variety and abundance of terpenes, with β-caryophyllene the most concentrated terpene, ranging from 25–42 mg/g (Figure 1 and Table S1). CO_2_ extracts displayed contents of primary terpenes such as β-caryophyllene and humulene but at lower concentrations compared to hydrodistilled samples. Ethanol extracts showed the least diversity in terpene composition, but contained amounts of geranyl acetate that were comparable to distilled extracts at a concentration range of 0.73–1.1 mg/g. No geranyl acetate was identified in CO_2_ extracts. Cannabinoid diversity was more prevalent within the CO_2_ extracts, and displayed all six targeted cannabinoids (Figure 2 and Table S2). CO_2_ extracts also contained the most CBD and CBG ranging from 12.8–20.6 mg/g and 9.5–16.9 mg/g, respectively. Ethanol extracts contained the most CBDA ranging from 3.2–4.1 mg/g and CBGA around 0.1 mg/g across all extraction types. CBD and CBC were the only identifiable cannabinoid within distilled extracts at concentrations less than 1 mg/g. Furthermore, each technique generated contrasting yields of extract mass with ethanol providing the greatest yield between 22–24%, CO_2_; 1.3–1.8%, and distilled, 0.08–1.1% (Table 1).

### 2.2. Statistical Analysis of Feature Relative Abundance

**Figure 3:**
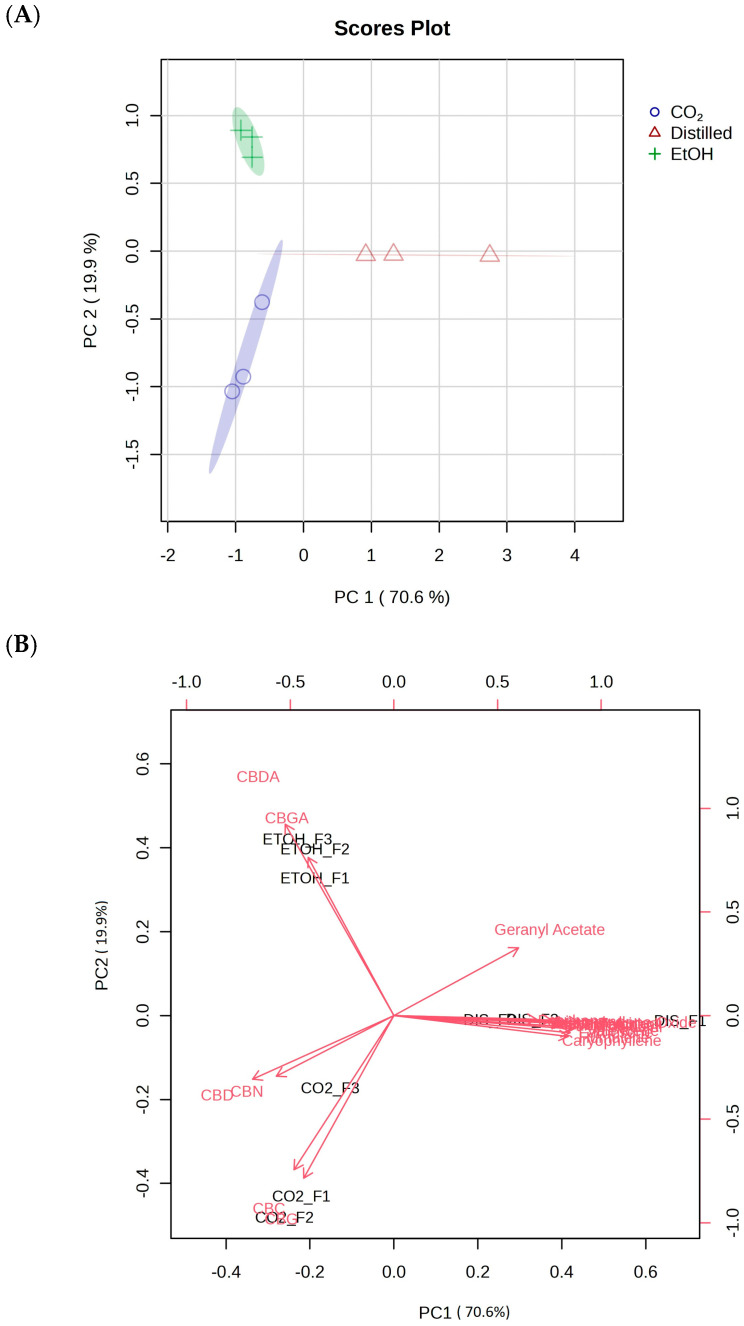
(**A**) Principal component analysis (PCA) plot of samples based on peak area of identified metabolites. (**B**) Biplot of samples with identified metabolite peak area as variables.

**Figure 4:**
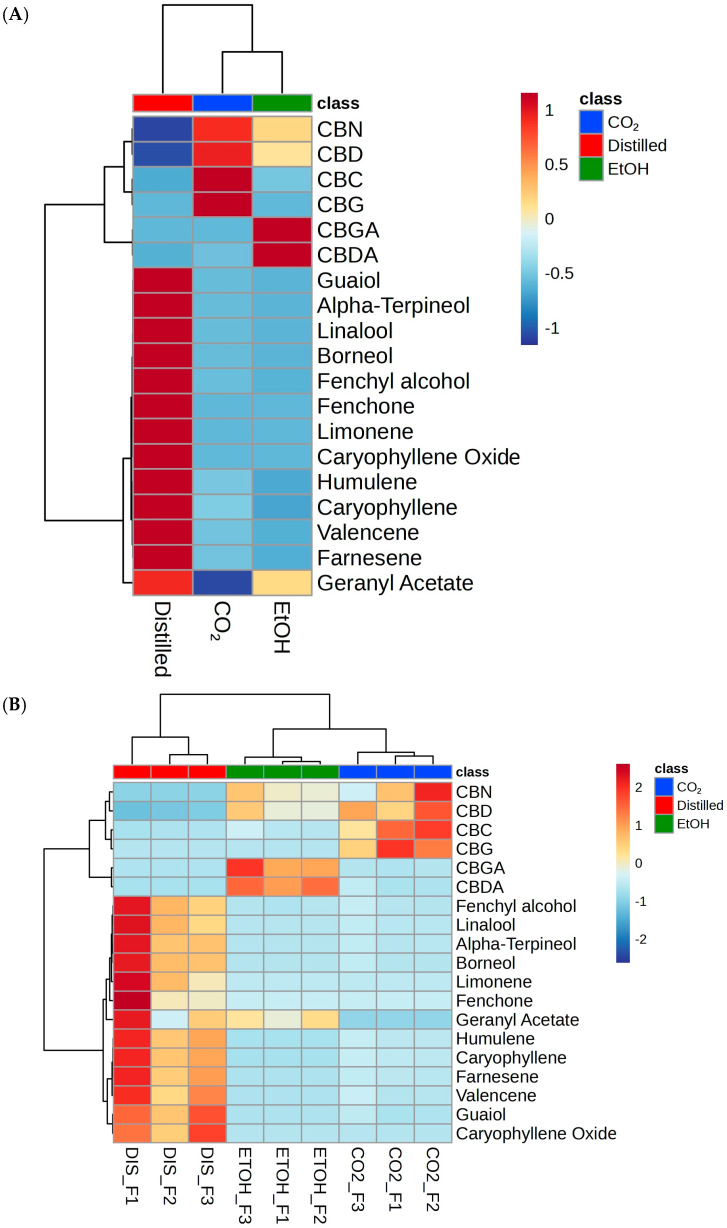
Heat map depicting the relative abundance of identified metabolites to samples. (**A**) average of triplicate strains per extraction technique. (**B**) individual samples across each extraction method. Samples are plotted as columns while identified metabolites are plotted as rows. Heatmaps created using MetaboAnalyst 6.0.

Principal component analysis (PCA) of the 19 identified compounds revealed a distinct clustering of the extraction techniques regardless of hemp sample (Figure 3A). The use of PCA reduces the dimensionality of our dataset while preserving crucial variance in the data structure, thus allowing for a better visualization and interpretation of the data. In an unsupervised setting, two principal components based on peak area displayed the clustering of samples with identical extraction techniques based on similar compounds. CO_2_ and ethanol samples demonstrated a tight cluster between each technique while a larger variation in the distilled samples resulted in a slight overlap in confidence level with CO_2_ extracts. The PCA biplot revealed that neutral cannabinoids had a higher correlation to CO_2_ samples while cannabinoid acids CBDA and CBGA correlated with ethanol extractions (Figure 3B). Terpenes showed a more variable correlation; however, most of the identified terpenes were present within the hydrodistillation samples with the exception of geranyl acetate which was also found in the ethanol extracts. These patterns were also observed in the heat maps displaying the relationship between specific compounds and samples. Heat map (Figure 4A) displayed the phytochemical landscape of compounds based on averages across extraction type. 

The acidic cannabinoids CBDA and CBGA were predominantly found within ethanol extracts, while the CO_2_ extract contained the neutral cannabinoids CBC, CBN, CBD, and CBG. At an individual level, slight variability was evident due to phytochemical differences between F1, F2, and F3 (Figure 4B). Ethanol samples displayed similar compound distributions between F1, F2, and F3. Distilled extracts’ terpene composition varied between samples, with F1 displaying a high concentration compared to F3 and F2, with the exception of guaiol and caryophyllene oxide being more concentrated in F3 distilled samples. CO_2_ extracts clustered together in general with greater relative abundance of neutral cannabinoids, though there were still differences between hemp source, with high significance between; CBC and CBG for F1, and all neutral cannabinoids for F2.

## 3. Discussion

The differences in cannabinoid and terpene composition between the extraction methods demonstrate the varying chemical profiles a single hemp variety can generate. As expected, the difference in polarity of the solvents often drives the variation we see in the resulting extract. Super critical CO_2_ extraction proved to be the most efficient type of extraction for both neutral cannabinoids and primary terpenes of the hemp cultivar. Distilled extracts yielded primary and secondary hemp terpenes but were significantly lower in the diversity and concentration of cannabinoids. Ethanol extractions provided an approach capable of pulling out cannabinoid acids with moderate terpene presence. Comparable amounts of geranyl acetate between distilled and ethanol extracts suggest that ethanol may be an optimal solvent for the extraction of terpenoids containing monocarboxylic acids. However, one investigation evaluating ethanol extracts differing in solvent temperature has shown evidence of a decrease in overall terpene content in extracts charged with room temperature ethanol compared to −40 °C ethanol [42]. This suggests that differences in terpene content evaluated in this study could be dependent upon the conditions that were applied. However, common across the three extraction techniques was their ability to extract CBD at various concentrations. One distinct difference between the processes of the super critical CO_2_ and solvent (ethanol) extractions is the added step in the CO_2_ extraction of decarboxylating the extract. This typically converts the acidic cannabinoids (e.g., CBDA and CBGA) into their neutral forms (CBD and CBG, respectively) [7,10]. Thus, as expected, the ethanol extraction yielded a higher amount of acidic cannabinoids which would otherwise be lost during the decarboxylation step of the supercritical CO_2_ extraction. Due to the nature of the decarboxylation process (i.e., the high heat (ca. 95 °C) for a long duration (~60 min)), this would not be readily amenable to ethanol-based extracts. Thus, this highlights another difference between these two processes that may make one more suitable over another for different research or commercial purposes.

It is important to note that this study provides evidence of unique cannabinoid and terpene profiles obtained through the three different extraction techniques at discreet conditions, and thus it is reasonable to assume that altering various parameters of an extraction protocol may yield further modification of the phytochemical profile obtained. Several studies have evaluated the efficiency of SFE extraction under varying conditions of pressure, temperature, and solvent consumption; however, results are also dependent on the specific cultivar [43,44,45]. Increasing the total amount of plant material and minimizing the storage time may likely result in higher yields and possibly more diversity in terpenes and cannabinoids for the specific hemp cultivar. Additionally, modification of the ethanol extraction process may also lead to differences in extract composition. In this study we make use of a simplified liquid solvent extraction approach that is easily reproducible and frequently employed at the industrial scale [46]. Previous publications involving ethanol extracts of cannabis species have observed that changes in solvent temperature and the minimizing pulverization can result in differences in compound abundance and extraction yield [42,47]. Although ethanol is excellent at drawing out bioactive compounds, and a primary method for other botanical supplement manufacturing processes, other compounds may also be present within the extract such as pigments from the plant material, limiting the selectivity. When considering hydrodistillation, one must consider the plant stage and handling process as both of these can influence the composition of terpenes at the time of extraction [48,49]. 

Furthermore, our approach of using two different analytical instrumentation methods (i.e., GC-MS and LC-MS/MS) revealed advantages for a potential multi-analytical approach to phytochemical analysis of cannabis but also revealed limitations of commonly accessible instrumentation. The GC-MS methodology employed in this investigation captured a range of different terpenes including a few that were observed after derivatization via trimethylsilyl addition. However, this analysis faced challenges as the GC-MS was equipped with only a single quad mass analyzer and only recorded low-resolution MS1 data; this was not sufficient to separate out some of the terpenes which have similar molecular composition and structure. Future work to compare chemical profiles of hemp extracts will utilize a GC analysis paired with tandem mass spectrometry allowing for better identification and capture of aromatic compounds [50]. On the other hand, LC-MS/MS analysis provided an advantage thanks to its additional fragmentation data, yet an LC approach made it difficult to capture all volatile compounds present within the extract. An untargeted approach may also be favorable for the identification of additional compounds that were not addressed in this investigation that may play a major role in the chemical and potential biological differences between extraction techniques.

## 4. Materials and Methods

### 4.1. Hemp Material and Solvents

A CBG/CBD dominant hemp cultivar “Tangerine” was grown, harvested, and donated by Cedar Meadow Farms, under three different field conditions and locations (hereafter referred to as F1, F2, and F3) (Holtwood, PA, USA). As a commercial hemp cultivar, the material is required to possess <0.3% THC by USDA regulation; therefore, THC and THCA standards were not included in this investigation. The inflorescence of each sampling that was used for extraction comparison were received 1–3 months prior to extraction and stored in vacuumed sealed bags at room temperature with limited light exposure. Once extracted, distilled samples were stored at 4 °C, ethanol extracts at room temperature, and super-critical CO_2_ extracts were stored at room temperature, all extracts were stored in amber glass vials. All solvents were acquired through VWR (Radnor, PA, USA) and were of reagent or spectroscopic grade. Standards were provided by Cayman Chemical (Ann Arbor, MI, USA) and confirmed certified reference materials. The processing of hemp material was done in accordance with and permitted by the Pennsylvania Department of Agriculture (permit #42-001074). 

### 4.2. Solvent Extraction

Methods for solvent extraction adapted and modified according to previously published protocols Kellogg et al., 2024 [51]; Szalata et al., 2022 [52]. For solvent extractions, 1.0 ± 0.05 g of inflorescence material was ground to a fine powder using an electric spice grinder and transferred to a 50 mL conical tube, after which 20 mL of 96% aqueous ethanol was introduced to each tube. Samples were vortexed for 10 sec then sonicated at room temperature (RT) for 30 min. Samples were centrifuged at 4 °C for 5 min at 3000 rpm, and the supernatant transferred to a clean 25 mL volumetric flask. The extraction was repeated two additional times to reach final volume of 25 mL of supernatant. The combined supernatant was vacuum filtered on a Buchner funnel with No. 1 Watman paper. The collected supernatant was then placed under −20 °C conditions for 24 h, filtered, then transferred to a scintillation vial and allowed to dry at RT under N_2_. Each field sample was extracted in triplicate.

### 4.3. Hydrodistillation

Distillation was carried out using 30 g whole flower samples. The method was adapted using protocols from Abraham et al., 2023 [50]. Each sample was placed inside a 1000 mL round bottom flask, after which 600 mL of Milli-Q water was charged. The sample was then placed on a heating mantel and attached to a distilling apparatus (VWR, Radnor, PA, USA). Samples were brought to a boil then lowered to a medium temperature for a total of 60 min. Oil from the return tube of the distillation apparatus was collected from the discharge stopcock into a 20 mL scintillation vial. For complete removal of water remnants, each oil was filtered using a glass pipette containing a loosely packed layer of glass wool below 0.8 g of anhydrous sodium sulfate. The filtered oil was then captured in a tared 20 mL scintillation vial. All samples were extracted in duplicate except for a single sample with restricted mass (F3). There was insufficient material to allow for triplicate analyses of each sample; however, with three biological replicates, and the accuracy and repeatability of the instrumentation, we determined it was sufficient to allow for comparison across methodologies.

### 4.4. Supercritical CO_2_ Extraction

Dried hemp flower, 55–105 g, was ground and extracted as previously described, with slight modifications [53], using an SFT-SP1100 extraction system manufactured by Supercritical Fluid Technologies, Inc. (Newark, DE, USA). Briefly, the extraction was performed at 55 °C for 35 min at 413 bars, the extract was then collected and dissolved in ethanol to a final concentration of 10% by weight. Winterization was carried out for 24–48 h at −20 °C and the extract was filtered before ethanol evaporation. The final extract was dissolved in fractionated coconut oil (Pure Body Naturals, West Chester, OH, USA) at 300 mg/mL and decarboxylated at 95 °C for 1 h.

### 4.5. LC-MS/MS

All samples were reconstituted at 50 µg/mL in methanol containing 1 µM of chlorpropamide as an internal standard (Sigma Aldrich, St. Louis, MO, USA), except for the distilled extracts which were prepared at 1 mg/mL. Standards were prepared as a serial dilution from 30–0.003 µg/mL. The method was adapted from Anderson et al., 2024 [54]. Samples were injected onto an Acquity UPLC BEH Shield RP18 (1.7 µm, 2.1 mm × 100 mm) column (Waters Corporation, Milford, MA, USA) and eluted into a Thermo Orbitrap Exploris 120 (ThermoFisher Scientific, Waltham, MA, USA) with an H-ESI ion source in the negative polarity mode and set to a scan range of 300–400 *m*/*z* with a resolution of 120,000. Samples were injected at a volume of 5 µL with the following binary system of solvent A, 0.1% formic acid in water, solvent B, 100% acetonitrile, at the following gradient: 50% B for 9 min, 100% B for 2 min, then 50% B for 2 min. The mass spectrometer was operated with a spray voltage of 2500 V, vaporizing temperature of 350 °C, aux gas of 10 Arb, sheath gas of 50 Arb, sweep gas of 1 Arb, and a collision gas pressure of 1 mTorr.

### 4.6. GC-MS

Samples were prepared at a concentration of 1 mg/mL in GC-MS grade hexane with 1 µM of chlorpropamide. After being transferred to GC-MS vials, they were allowed to dry under a fume hood with open covers to prevent contamination of samples. Once dried, samples were introduced to pyridine and BSTFA (1:1), capped and allowed to sit for 60 min at room temperature to allow for the addition of a trimethylsilyl group for increased compound separation. Samples were then stored at −20 °C until MS analysis. Terpenoid standards were prepped in a similar manner but at a concentration range of 500 µg/mL–0.3 µg/mL. Cannabinoid standards were prepared at a concentration of 50 µg/mL for identification. Analysis of the samples and standards were carried out using methods adapted from Abraham et al., 2023 [50] on an Agilent 5975C series GC-MSD with an Electron Ionization (EI) source operated in the positive mode with a scan range of 50–600 *m*/*z* [50]. All samples were injected at 1 µL on a 30 m × 250 µm × 0.25 µm Restek 13423 column (Restek, Bellefonte, PA, USA), which was initially set to 50 °C and pressure of 7.6522 psi. The oven temperature was ramped at 4 °C/min rate until 280 °C with a hold time of 2 min (61.5 min total run time).

### 4.7. Data Analysis

Compounds of interest in the GC-MS samples were identified based on MS1 data of the involved standards visualized in MassHunter Qualitative Analysis Agilent software (Qualitative Analysis 10.0). LC samples were identified using the MS2 data in MZMine3 [34,55]. Peak area measurements were accomplished using MZMine3 for GC samples, while measurements for LC samples were determined by Thermo Xcalibur Quantitation Analysis (ThermoFisher Scientific). MZmine Workflow parameters and standard concentration curves can be found within the Supplementary Materials. MetaboAnalyst 6.0 was used for principal component analysis (PCA), loading plot, and heat map construction (www.metaboanalyst.ca (accessed on 6 August 2024)). Relative abundance of identified features were used independent variables for each statistical analysis with a range scaling normalization of the data for the PCA. Equation (1) was used to quantify individual compounds, where “concentration of compound” was the calculated amount injected based on the corresponding calibration curves, “extraction vol/sample aliquot” was the sample preparation dilution. Equation (2) determined total extraction yield (%), based upon the ration between the dried extract mass and the dried plant material (inflorescence) mass. Extraction yields for distilled samples were limited to one replicate due to weight limitations.

(1) $$Compound\frac{mg}{g}\left(\%\right)=\left(\begin{array}{c} \mathrm{Concentration}\\ \mathrm{of}\;\mathrm{compound},(\frac{\mu g}{mL}) \end{array}\right)\left(\frac{extraction\;vol.(mL)}{sample\;aliquot,\;(mg)}\right)\left(\begin{array}{c} \mathrm{conversion}\;\\ \frac{\mu g\;to\;mg}{mg\;to\;g} \end{array}\right)\times 100$$

(2) $$Extracton\;Yield\left(\%\right)=\left(\frac{Extract\;Dry\;Mass\;(mg)}{Inflorescence\;dry\;Mass\;(mg)}\right)\times 100$$

## 5. Conclusions

Overall, this study provides evidence of the differences in cannabinoid and terpene composition obtained from hemp inflorescence after three predominant extraction techniques: supercritical CO_2_, solvent extraction (ethanol), and hydrodistillation. While supercritical CO_2_ extraction can achieve broad extraction and has a relatively short extraction time of large quantities of plant material, a supercritical CO_2_ extractor is expensive. Challenges with solvent extractions involving ethanol will likely make isolation of neutral cannabinoids difficult and result in a more diverse phytochemical profile. Likewise, hydrodistillation can be extremely effective at capturing terpenes within hemp inflorescences; however, the process has dramatically lower yields and a distinct lack of cannabinoids in the resulting extract. The results from this head-to-head investigation with identical plant material demonstrate how extraction techniques can allow commercial processors or academic investigators to be more selective in the extraction process and fine-tune their processes to develop more specific hemp extracts.

## Figures and Tables

**Table 1:** Extraction yield % ± SD (*w*/*w*) of different extraction techniques across three hemp samples.

	F1			F2			F3		
	Distilled	Ethanol	CO_2_	Distilled	Ethanol	CO_2_	Distilled	Ethanol	CO_2_
Extraction yield *w*/*w* (%)	1.11	24.29 ± 0.02	1.84 ± 0.01	0.18	22.03± 0.01	1.56 ± 0.01	0.08	24.27 ± 0.01	1.30 ± 0.00

## Data Availability

The mass spectral datasets from this study can be found in the MASSive data repository (https://doi.org/10.25345/C5DZ03C6Z).

## Supplementary Materials

The following supporting information can be downloaded at: https://www.mdpi.com/article/10.3390/plants13162222/s1, Table S1: Mean concentration of identified terpenes ± SD (mg/g) in sample extract; Table S2. Mean concentration of identified cannabinoids ± SD (mg/g) in sample extract; Figures S1–S6: Cannabinoid calibration curves; Figures S7–S19: terpene calibration curves; csv file of GC feature list summary.
