# Supplementary material for: Effect of Hemp Extraction Procedures on Cannabinoid and Terpenoid Composition
Source: Plants (Basel). 2024 Aug 10;13(16):2222. doi: 10.3390/plants13162222 (PMC11359220; doi:10.3390/plants13162222)
Supplement: Supplementary file 1 [file plants-13-02222-s001.zip › Supporting_Information.pdf]

Supporting Information

Effect of Hemp Extraction Procedures on Cannabinoid and Terpenoid Composition

Francisco T. Chacon[1], Wesley M. Raup-Konsavage [2], Kent E. Vrana [2], and Joshua J. Kellogg [1,3,*]

[1] Intercollege Graduate Degree Program in Plant Biology, Pennsylvania State University, University Park, State College, PA 16802, USA

[2] Department of Pharmacology, Penn State College of Medicine, Hershey, PA 17033, USA

[3] Department of Veterinary and Biomedical Sciences, Pennsylvania State University, University Park, State College, PA 16802, USA

*** Correspondence:** jjk6146@psu.edu; Tel.: +1-814-865-2887

Supplementary Data

Supplemental Table S1. Mean concentration of identified terpenes ± SD (mg/g) in sample extract. Limit of quantification (LOQ) and not detected (ND).

Terpene (mg/g)	F1			F2			F3		
	**Distilled**	**Ethanol**	**CO_2**	**Distilled**	**Ethanol**	**CO_2**	**Distilled**	**Ethanol**	**CO_2**
**β-Farnesene**	1.878 ± 0.014	ND	0.313 ± 0.000	0.933 ± 0.002	ND	0.131 ± 0.002	1.220	ND	0.409 ± 0.000
**Valencene**	1.995 ± 0.011	ND	0.048 ± 0.000	0.798 ± 0.002	ND	0.097 ± 0.001	1.423	ND	0.210 ± 0.001
**(1R)-endo(+)-Fenchyl alcohol**	6.600 ± 0.069	0.100 ± 0.002	0.475 ± 0.001	4.095 ± 0.001	0.431 ± 0.003	0.185 ± 0.003	3.205	0.261 ± 0.002	0.886 ± 0.001
**Humulene**	8.580 ± 0.056	ND	1.757 ± 0.001	5.447 ± 0.007	ND	1.751 ± 0.005	6.759	ND	2.158 ± 0.003
**Guaiol**	9.472 ± 0.046	0.117 ± 0.001	0.044 ± 0.001	6.753 ± 0.014	0.146 ± 0.001	0.278 ± 0.001	12.458	0.212 ± 0.000	0.971 ± 0.004
**β -Caryophyllene**	42.717 ± 0.323	ND	4.020 ± 0.008	25.138 ± 0.052	ND	2.673 ± 0.045	31.569	ND	5.664 ± 0.020
**Caryophyllene oxide**	25.973 ± 0.010	ND	ND	15.089 ± 0.013	ND	1.750 ± 0.010	31.380	ND	2.047 ± 0.002
**Linalool**	1.738 ± 0.016	ND	<LOQ	0.831 ± 0.000	ND	0.012 ± 0.000	0.581	ND	0.064 ± 0.000
**α-Terpineol**	3.793 ± 0.027	ND	<LOQ	1.713 ± 0.000	ND	0.393 ± 0.000	1.713	ND	0.245 ± 0.001
**Fenchone**	0.124 ± 0.002	ND	ND	<LOQ	ND	ND	<LOQ	ND	ND
**Geranyl Acetate**	2.587 ± 0.009	0.732 ± 0.014	ND	0.359 ± 0.001	1.064 ± 0.005	ND	1.119	0.844 ± 0.011	ND
**Limonene**	4.929 ± 0.036	ND	ND	3.064 ± 0.006	ND	ND	2.315	ND	ND
**Borneol**	1.358 ± 0.011	ND	ND	0.612 ± 0.000	ND	ND	0.582	ND	<LOQ

Supplemental Table S2. Mean concentration of identified cannabinoids ± SD (mg/g) in sample extract. Not detected (ND).

Cannabinoids (mg/g)	F1			F2			F3		
	Distilled	Ethanol	CO_2	Distilled	Ethanol	CO_2	Distilled	Ethanol	CO_2
**CBD**	0.95	9.32 ± 0.00	12.78 ± 0.01	1.41	9.24 ± 0.02	20.55 ± 0.07	1.74	13.57 ± 0.01	16.07 ± 0.05
**CBDA**	ND	3.23 ± 0.00	0.03 ± 0.00	ND	4.05 ± 0.01	0.06 ± 0.00	ND	4.13 ± 0.07	0.44 ± 0.01
**CBG**	ND	ND	16.89 ± 0.08	ND	ND	16.38 ± 0.05	ND	1.38 ± 0.01	9.54 ± 0.00
**CBGA**	ND	0.10 ± 0.00	0.01 ± 0.00	ND	0.11 ± 0.00	0.01 ± 0.00	ND	0.17 ± 0.00	0.01 ± 0.00
**CBN**	ND	0.49 ± 0.00	0.54 ± 0.00	ND	0.49 ± 0.00	0.63 ± 0.00	ND	0.53 ± 0.00	0.47 ± 0.00
**CBC**	0.01	0.17 ± 0.00	0.42 ± 0.00	0.02	0.17 ± 0.00	0.46 ± 0.00	0.02	0.20 ± 0.00	0.26 ± 0.00

Cannabinoid calibration curves

Linear calibration curves for six cannabinoids standards at a range of 30 – 0.003 ug/ml.

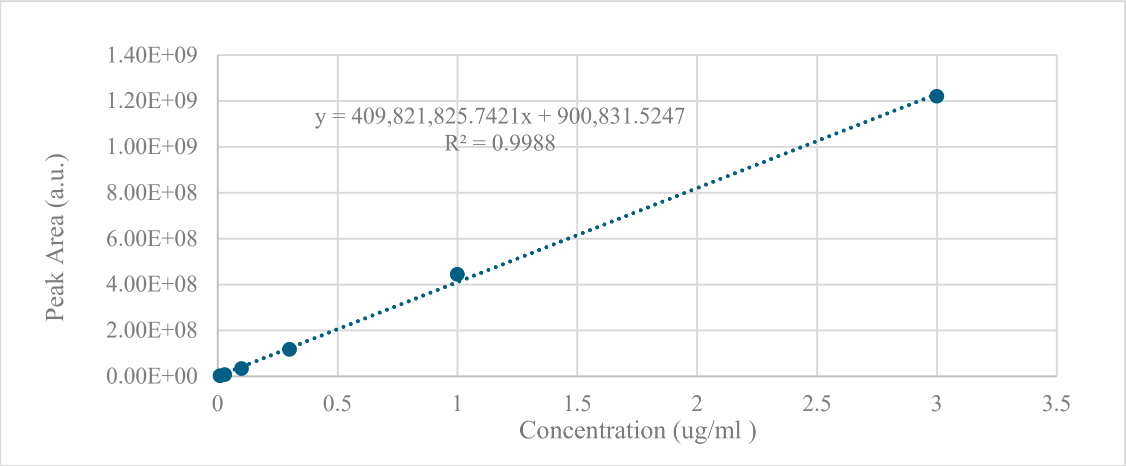

Supplementary Figure S1. Cannabidiolic acid (CBDA) calibration curve.

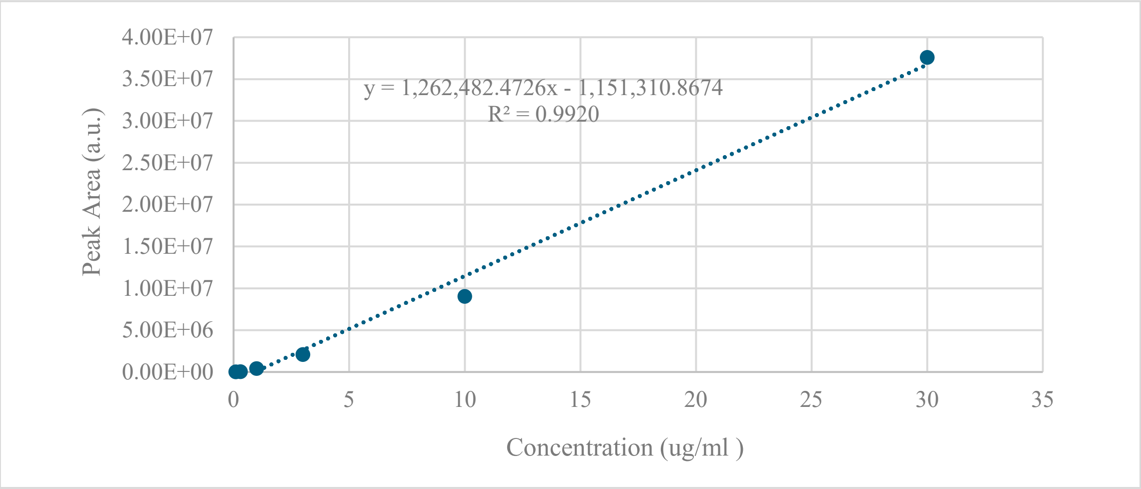

Supplementary Figure S2. Cannabidiol (CBD) calibration curve.

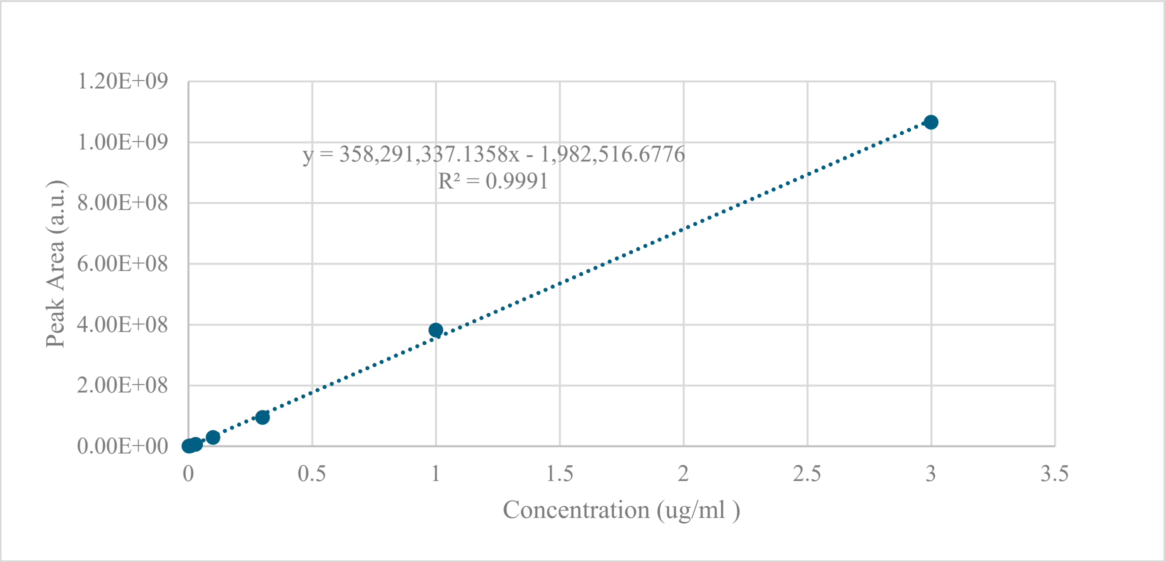

Supplementary Figure S3. Cannabigerolic acid (CBGA) calibration curve.

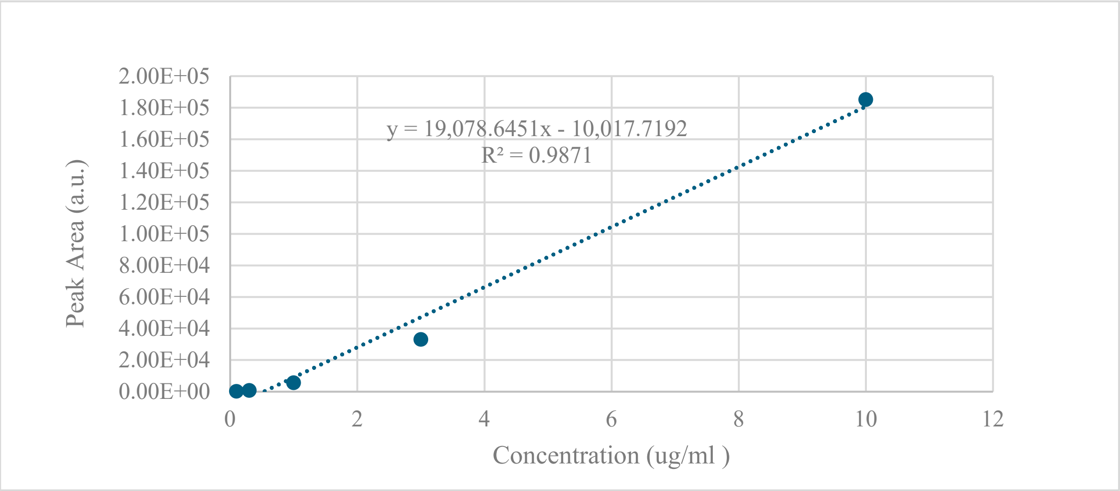

Supplementary Figure S4. Cannabigerolic acid (CBGA) calibration curve.

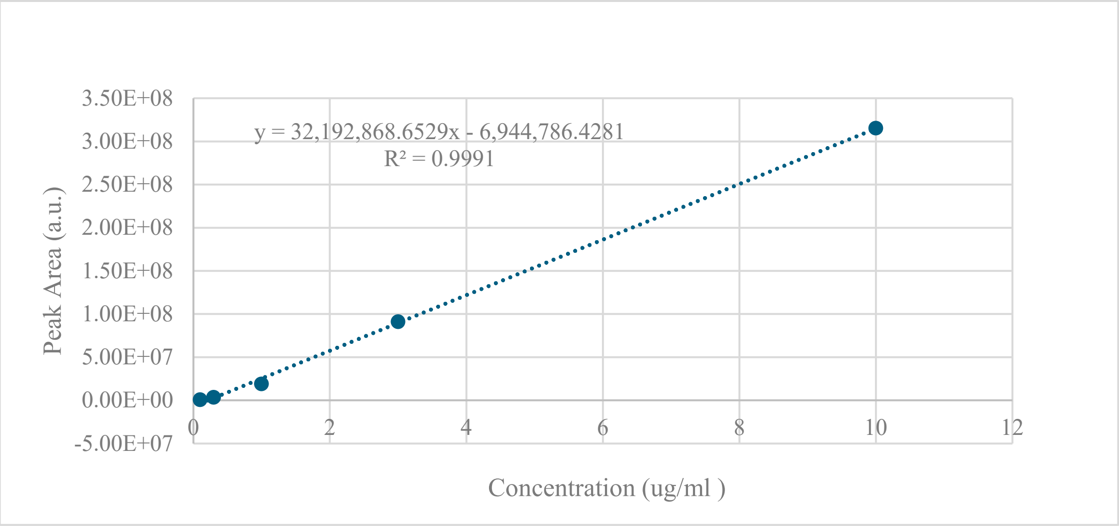

Supplementary Figure S5. Cannabinol (CBN) calibration curve.

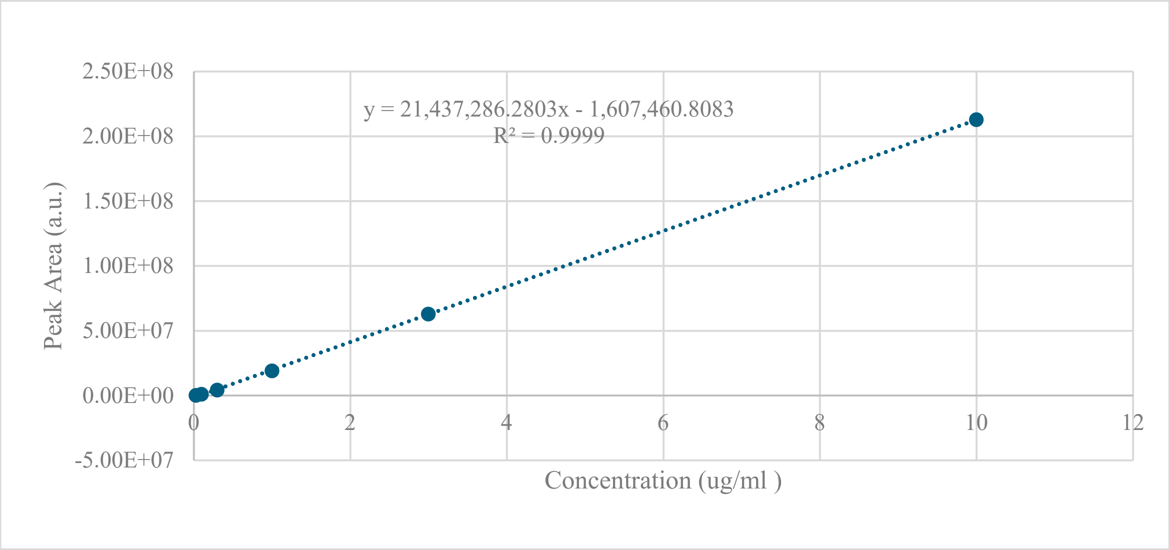

Supplementary Figure S6. Cannabichromene (CBC) calibration curve.

Terpene calibration curves

Linear calibration curves for 13 terpene standards at a range of 50 ug/ml – 0.003 ug/ml

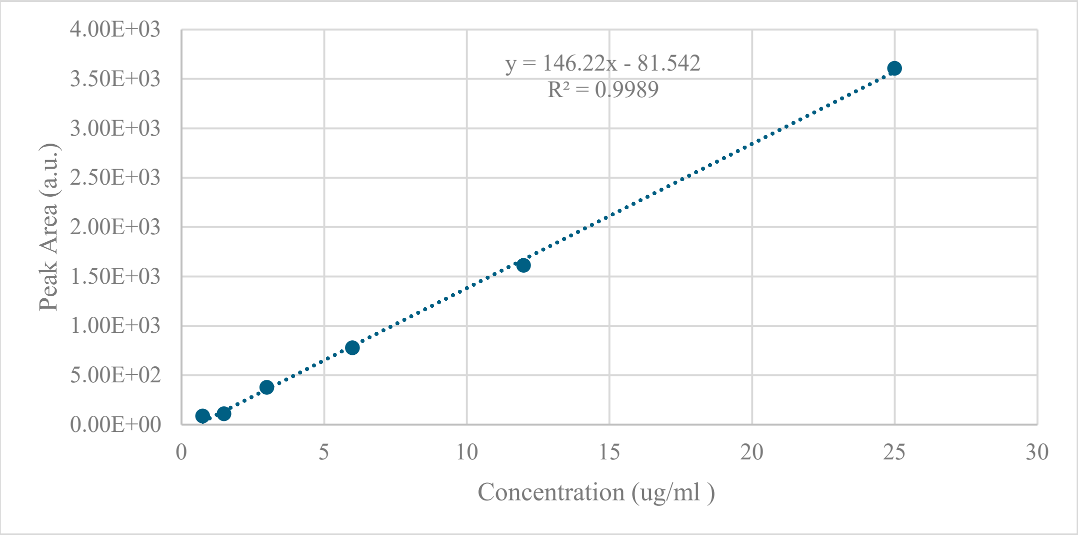

Supplementary Figure S7. Fenchone calibration curve.

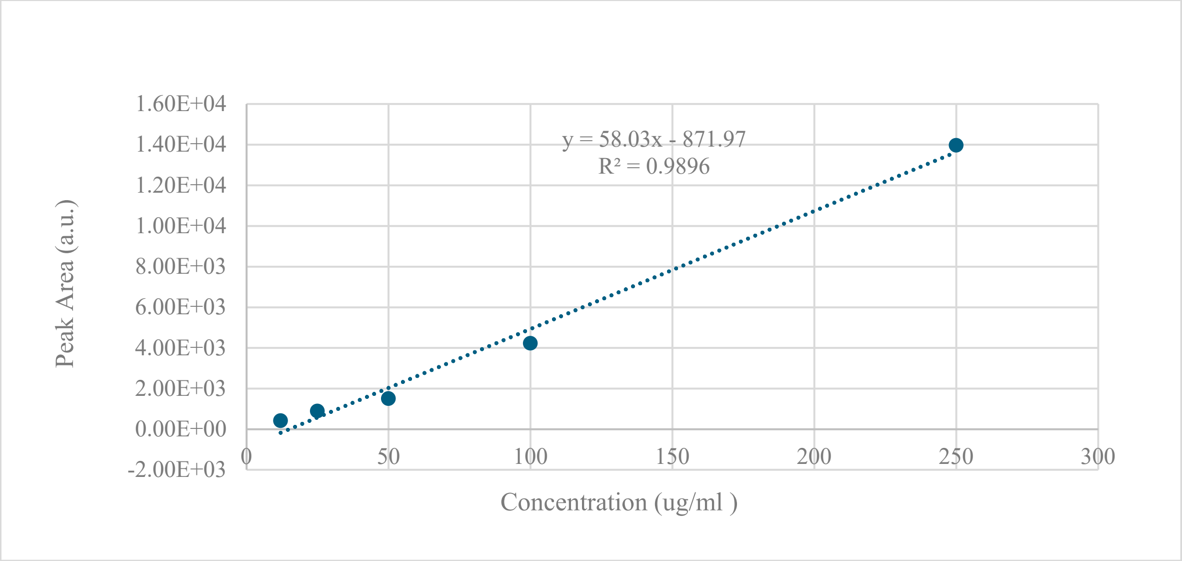

Supplementary Figure S8. Limonene calibration curve

.

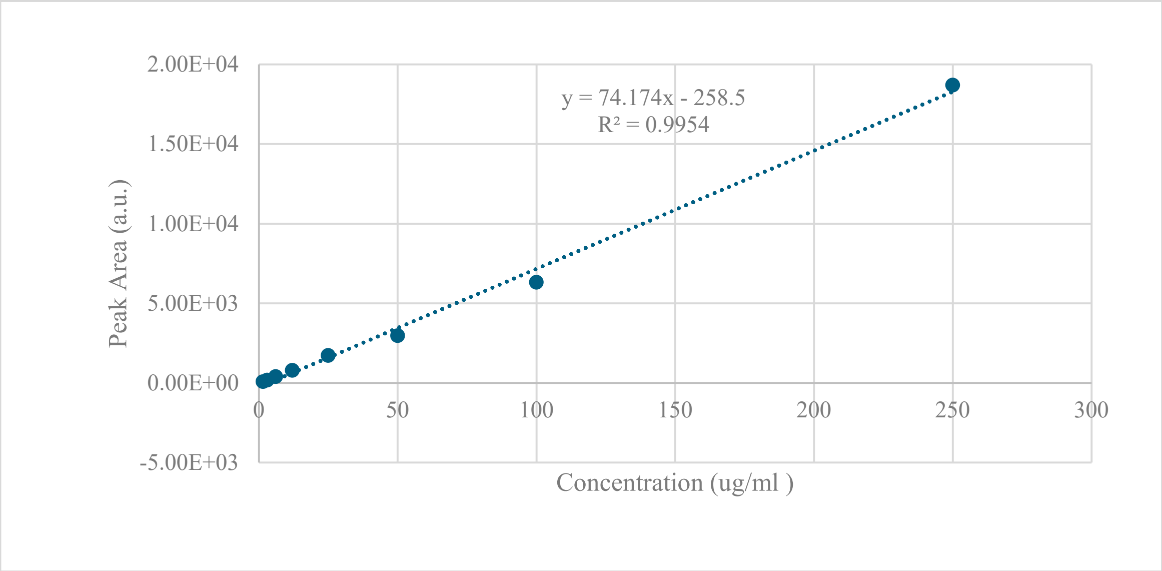

Supplementary Figure S9. endo(+)-Fenchyl alcohol calibration curve.

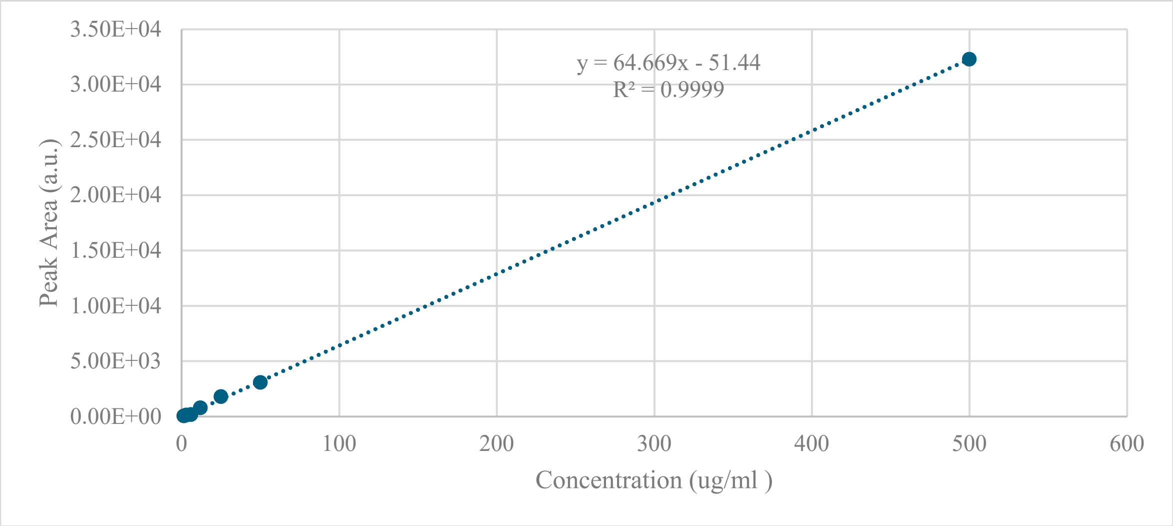

Supplementary Figure S10. β - caryophyllene calibration curve.

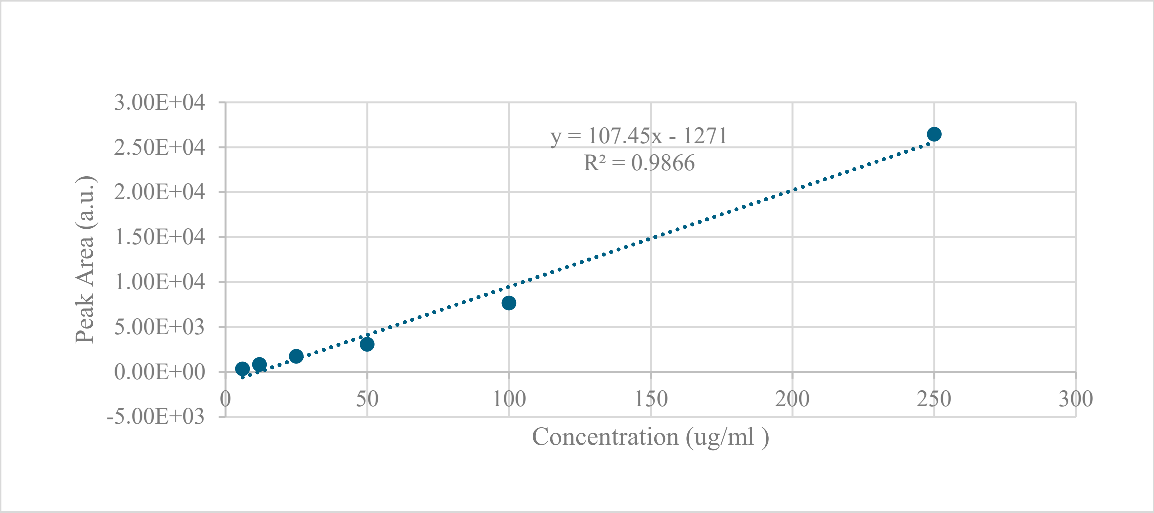

Supplementary Figure S11. Humulene calibration curve.

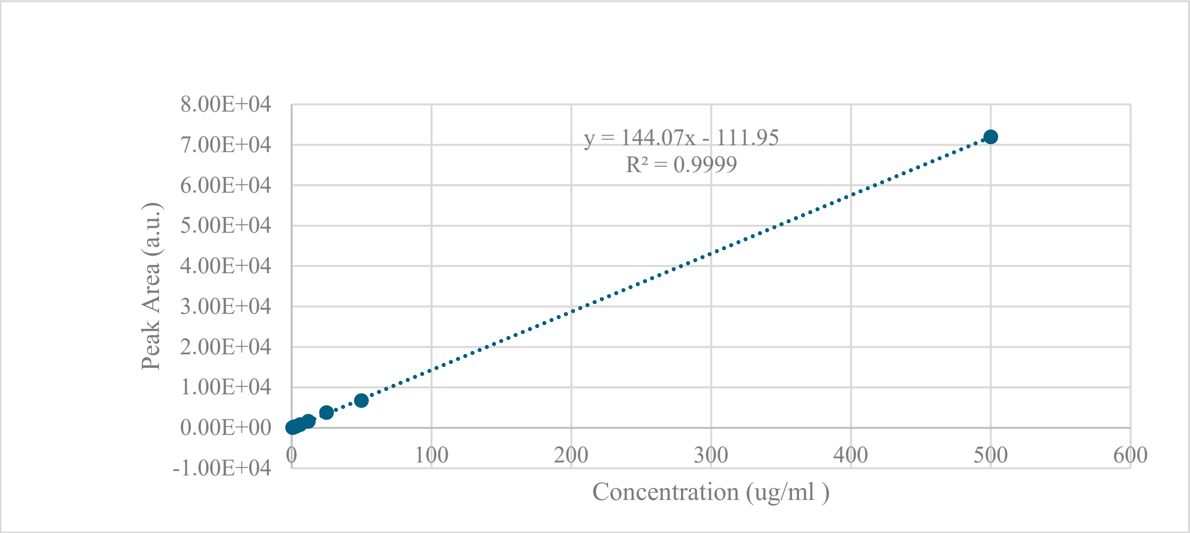

Supplementary Figure S12. Guaiol calibration curve.

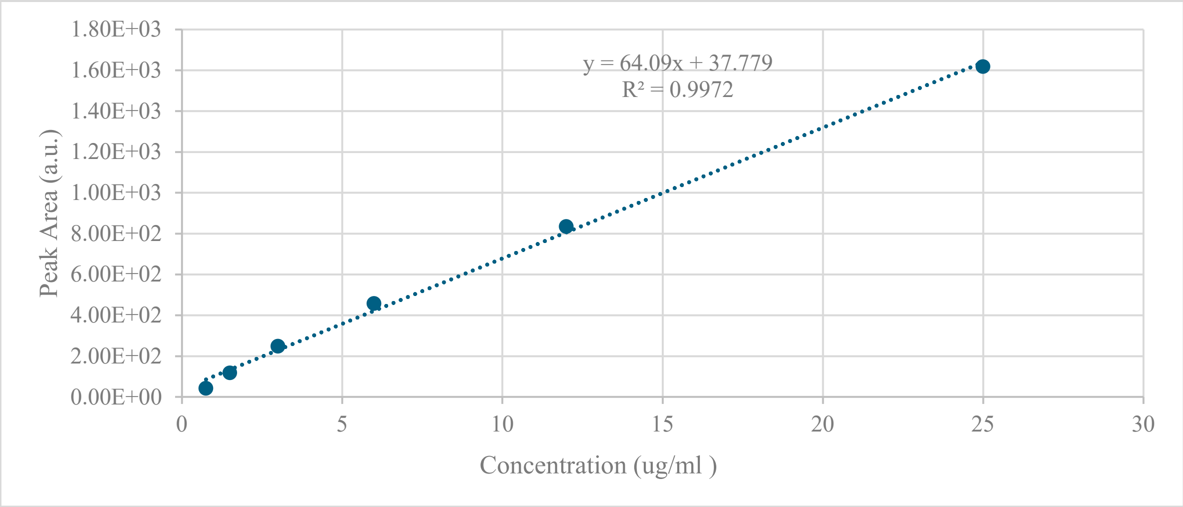

Supplementary Figure S13. Borneol calibration curve.

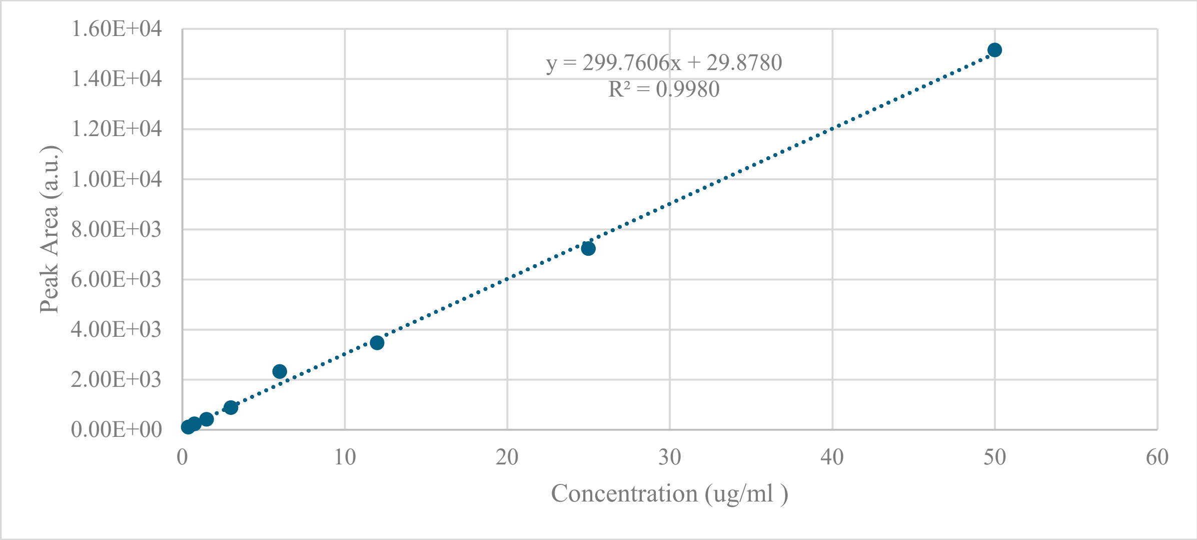

Supplementary Figure S14. Linalool calibration curve.

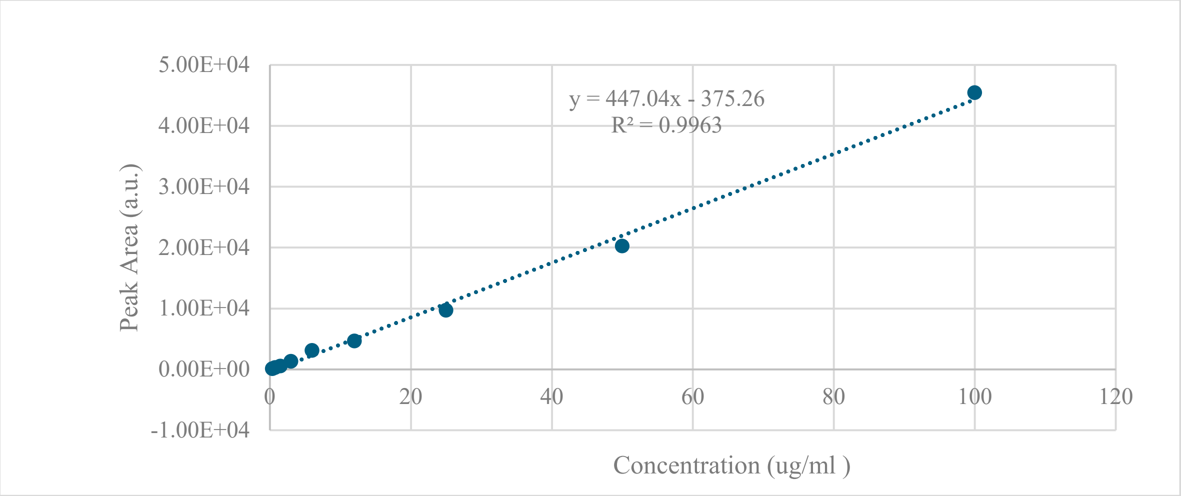

Supplementary Figure S15. Alpha-terpineol calibration curve.

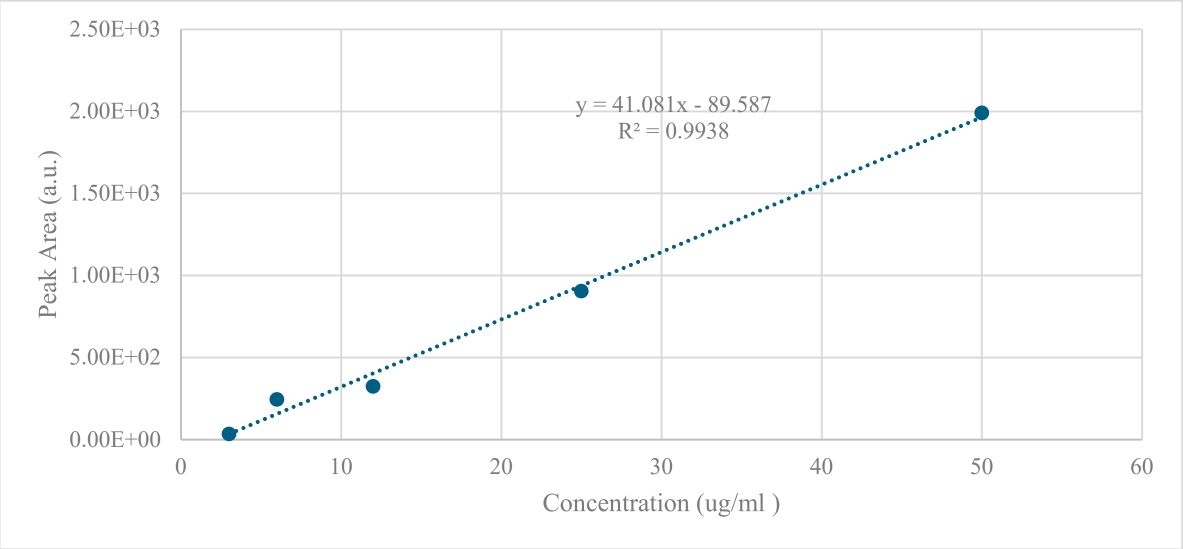

Supplementary Figure S16. Farnesene calibration curve.

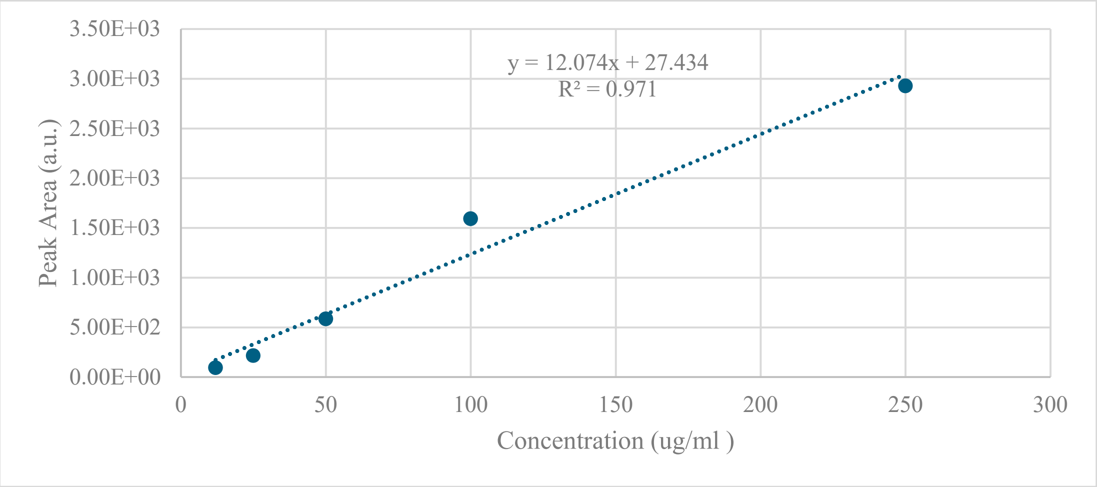

Supplementary Figure S17. Geranyl acetate calibration curve.

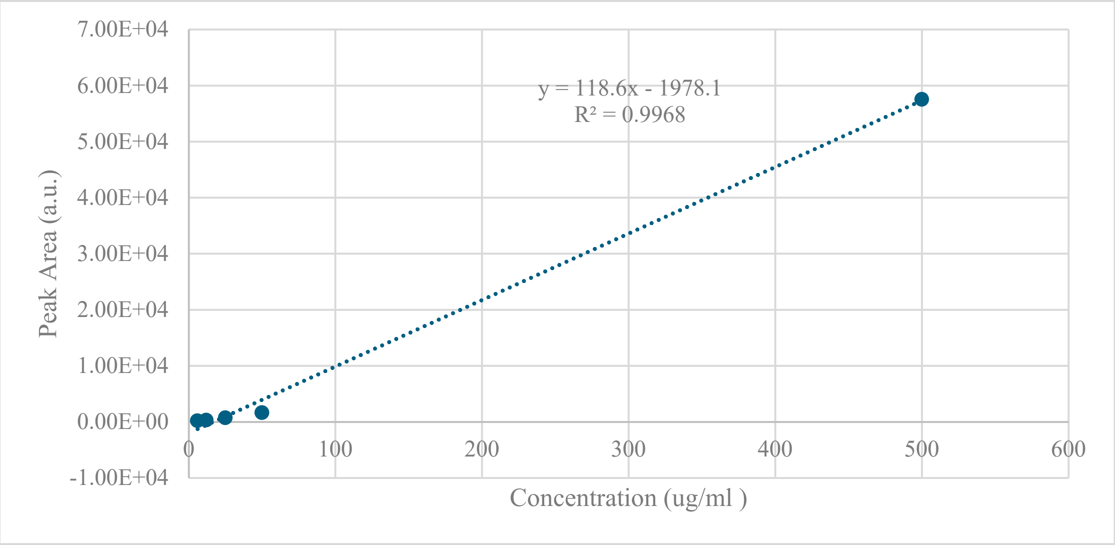

Supplementary Figure S18. Caryophyllene oxide calibration curve.

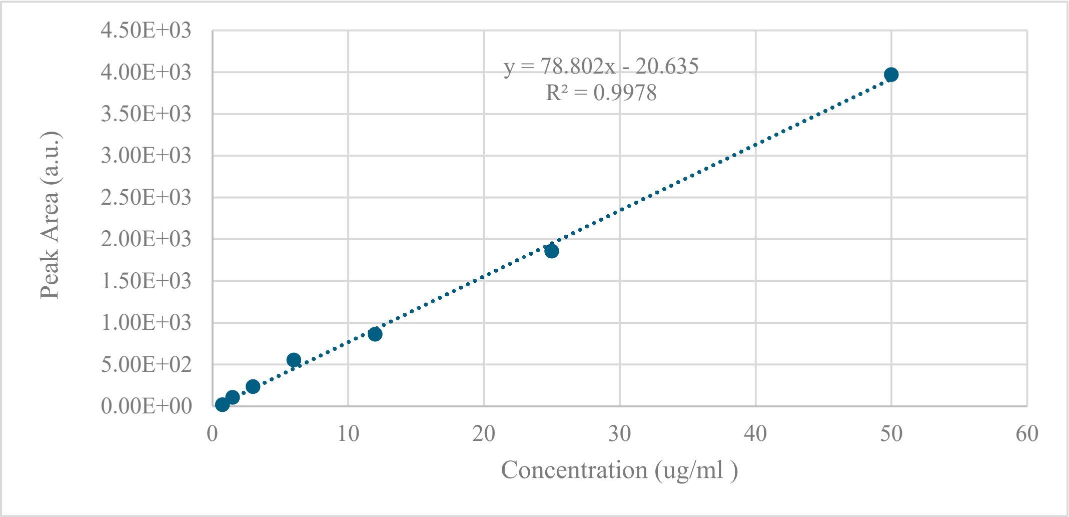

Supplementary Figure S19. Valencene calibration curve.
